# The Anti-Cancer Effect of Cinnamon Aqueous Extract: A Focus on Hematological Malignancies

**DOI:** 10.3390/life13051176

**Published:** 2023-05-12

**Authors:** Santino Caserta, Claudia Genovese, Nicola Cicero, Sebastiano Gangemi, Alessandro Allegra

**Affiliations:** 1 Division of Hematology, Department of Human Pathology in Adulthood and Childhood “Gaetano Barresi”, University of Messina, Via Consolare Valeria, 98125 Messina, Italy; 132588@polime.it (S.C.); aallegra@unime.it (A.A.); 2 National Research Council, Institute for Agricultural and Forest Systems in the Mediterranean, Via Empedocle 58, 95128 Catania, Italy; 3 Department of Biomedical, Dental, Morphological and Functional Imaging Sciences, University of Messina, Via Consolare Valeria, 98125 Messina, Italy; ncicero@unime.it; 4 Allergy and Clinical Immunology Unit, Department of Clinical and Experimental Medicine, University of Messina, Via Consolare Valeria, 98125 Messina, Italy; gangemis@unime.it

**Keywords:** cancer, cinnamon, hematological malignancies, leukemia, lymphoma, angiogenesis, immune response, inflammation, cell proliferation, apoptosis

## Abstract

Cinnamon is an evergreen and tropical plant of the family *Lauraceae*, growing particularly in Sri Lanka, whose aqueous extract has been tested in different studies to evaluate its possible use as an anti-cancer compound. Both in vitro and in vivo experiments seem to confirm that it acts on various cellular pathways, contributing to down-regulating the activity of molecules that stimulate the proliferation and survival of cells such as the transcription factors NF-KB and AP-1, COX-2, dihydrofolate reductase and pro-angiogenic substances such as VEGF, while up-regulating the function of immune cells against tumors, such as cytotoxic CD8+ T cells. In hematological malignancies, aqueous cinnamon extract has been studied in order to understand if it is possible to count on its help, alone or in combination with traditional drugs such as doxorubicin, to treat patients. The aim of our work is to investigate results from in vitro and in vivo studies about the possible anti-cancer effect of aqueous cinnamon extract in hematological malignancies and the different pathways involved in its action. The possibility of using cinnamon extract in clinical practice is discussed; even if its use could appear very interesting, more studies are necessary to clear the real potentiality of this substance in cancer.

## 1. Introduction

Cinnamon is an evergreen and tropical plant of the family *Lauraceae* [1], growing particularly in Sri Lanka [2], whose aqueous extract contains different substances such as the essential oils cinnamic aldehyde and cinnamyl aldehyde. The possibility of using natural substances as a cure is the fundamental of complementary and alternative medicines (CAMs), and cinnamon aqueous extract could have a crucial role in this field because of its anti-oxidant and anti-inflammatory effects [1]. Its range of activity is wide; in fact, cinnamon is used especially as a flavoring agent and a spice [3], and Sri Lankan medicine uses its extract from years to treat diabetes [4,5], allergies, microbial and parasitic diseases because of its action on different pathogens such as Mycobacterium tuberculosis and Streptococcus pneumoniae [6]. Cinnamon also has an anti-cancer effect due to the interaction of its molecules in different pathways involved in the proliferation, survival, spread and programmed death of cells [7]. The aqueous extract can be obtained from crushing cinnamon bark and leaving it to stand for three hours in hot water; it is filtered and frozen, becoming a sort of powder, and is finally mixed with sterilized water at a specific dose. The concentration of its principal compounds was measured, resulting in 2.9 mg/g of extract and 7.9 mg/g of extract of cinnamic aldehyde and cinnamyl aldehyde [1]. Studies also investigated the possible effects of aqueous cinnamon extract on hepatic and renal functions after administration of five, ten and twenty times the effective dosage of it in healthy mice and demonstrated that, comparing a group of rats treated with aqueous cinnamon extract with another one of untreated animals, after an administration of a dose twenty times higher than the effective one, a minimum augment in alkaline phosphatase and transaminases can be observed in rats which received the substance, but after seventy-two hours a normalization of these values occurs. As far as renal function is concerned, cinnamon extract determines a decrease in serum creatinine level and, in contrast, a rise in urea and uric acid levels compared with untreated rats [8]. This evidence lets us state that cinnamon extract could have a negative influence on hepatic function, while having both a positive and negative effect on kidney activity.

## 2. Cinnamon and Cancer: Pathways and Mechanisms of Action

Lifestyle, including diet, has a crucial role in the pathogenesis of cancer; in fact, it is well-known that the Mediterranean diet, based on regular hydration with above two liters of water daily; an adequate intake of vegetables, fruits, legumes and unrefined cereals rich in vitamins and antioxidants substances; and a minimum quantity of red meat and other foods, represents a valid protective factor against cancer [9,10].

Foods from vegetal world, plants and herbs are the object of different studies with goals to attempt to understand the mechanisms of interaction between vegetal molecules and human diseases. Aqueous cinnamon extract has been tested in breast cancer cells and it demonstrated to be able to up-regulate the function of specific genes such as the peroxisome proliferator-activated receptor (PPARG) [11]: it encodes for a nuclear receptor which, in breast cancer cells, has an essential role in homeostasis, cellular metabolism and neoplastic progression. In particular, PPARG acts via suppressing the proliferation and migration of cells and stimulating apoptosis. Studies put in evidence that, in patients with breast cancer, high levels of PPARG are related to a major survival rate compared with those with lower expressions of this nuclear receptor [12]. In prostate cancer cells, aqueous cinnamon extract has been tested as an anti-proliferative molecule and as a proteasome inhibitor. In fact, it is capable of selectively inhibiting the proliferation of cancer cells. Proteasome is a system of proteins which, through ubiquitination, regulates the proliferation and degradation of cells [13]; it is the target for drugs’ so-called proteasome inhibitors such as Bortezomib, used in the treatment of multiple myeloma and other tumors [14]. The use of natural substances, and in particular of aqueous cinnamon extract, as proteasome inhibitors in prostate cancer could represent a fascinating field of study and lead to new instruments for curing patients [15].

### 2.1. Inhibition of NF-KB and AP-1

**Figure 1:**
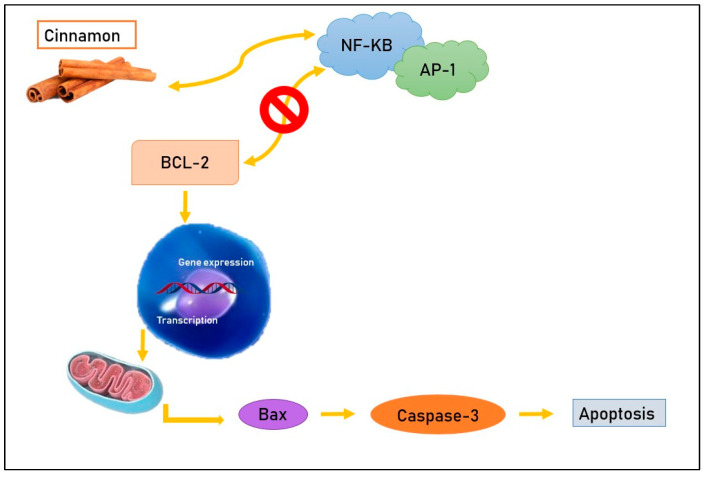
Cinnamon compounds tie to NF-KB and AP-1, preventing their bond with the anti-apoptotic protein Bcl-2 and leading, consequently, to apoptosis.

In vivo experiments [1] suggest that aqueous cinnamon extract acts on specific molecules such as the transcription factors nuclear factor kappa-light-chain-enhancer of activated B cells (NF-KB) and Activator protein 1 (AP-1), which have a crucial role in cell survival and in the regulation of the cell cycle and apoptosis. In fact, NF-KB and AP-1 have as a target the anti-apoptotic protein Bcl-2 [16] and, normally, cause an increase in this molecule with the consequent inhibition of programmed death of tumor cells [17]. Cinnamon compounds tie to NF-KB and AP-1, determining a decrease in the correspondent RNA expression and translation and preventing the anti-apoptotic action of Bcl-2, which they commonly stimulate [1] (Figure 1). In another in vivo study [9], mice in which melanoma cells (B16F10) were subcutaneously transplanted were divided into two subsets: in one group, animals received cinnamon extract daily at a dose of 400 μg/g of weight, and in the other group animals received phosphate-buffered saline (PBS). After thirty days of treatment, the tumor size was evaluated based on tumor weight and, interestingly, an important decrease was observed in the subset of mice that received cinnamon (tumor weight 6.2 g) compared with those that received PBS (tumor weight 12.2 g). These results show that cinnamon aqueous extract, acting on the transcription factors NF-KB and AP-1, has an important anti-cancer effect in vivo [1]. Even if aqueous cinnamon extract has not lead to morphological changes in cells 24 h from exposure, a progressive augment in apoptotic cells has been observed [18] and it seems that the apoptosis rate gradually increases depending on the time from cinnamon administration; in fact, in order to verify this condition, neoplastic cells of colorectal adenocarcinoma have been treated with this natural compound and showed a similar time-related pattern in increasing apoptosis rate. Furthermore, aqueous cinnamon extract proved to be capable of stimulating the expression of pro-apoptotic genes such as Bim and Bax [19,20,21,22,23,24,25,26].

### 2.2. Activation of Cytotoxic CD8+ T Cells

Cytotoxic CD8+ T cells have a fundamental function in the immune response against tumors and prevent the so-called “immune escape”; thanks to neoplastic cells, they are able not to be recognized as non-self-agents [18], but their activity is sometimes suppressed from the tumoral microenvironment or from alterations such as reduced expression of perforins, inefficacy of adhesion, abnormal production of cytokines and defective action of exocytosis granules [27,28,29]. CD8+ T cells, stimulated by ingredients in cinnamon extract, determine an increase in the expression of molecules with cytolytic action such as granzymes B, granzymes C, interferon-C and tumor necrosis factor-alpha (TNF-α) [18,30,31,32,33,34]. In detail, there is evidence in vitro of this effect in cells from breast cancer, colorectal cancer, melanoma and liver cancer [17]. It has been also demonstrated that there are differences between oral and subcutaneous administration of aqueous cinnamon extract; in fact, an oral treatment of more of 48 h inhibits the growth of tumor cells via apoptosis more strongly than that of a subcutaneous one through an important reduction in pro-angiogenic molecules and pro-inflammatory cytokines, while a subcutaneous administration determines a major activation of cytotoxic CD8+ T cells with an anti-cancer effect. These observations could indicate a possible effect of cinnamon extract as an anti-cancer molecule thanks to its capacity to recruit cytotoxic CD8+ T cells and potentiate their activity against neoplastic cells.

### 2.3. Inhibition of COX-2 and Inflammatory Cells

Inflammation has a crucial role as a defense mechanism against pathogens and other external agents. It starts after a trigger, for example a virus, activates a cascade of intracellular and intercellular signals, leading to the release of inflammatory molecules such as nitric oxide (NO), prostaglandins (PGs) and TNF-α [35]. This situation causes a disequilibrium between pro-inflammatory and anti-inflammatory cytokines with a prevalence of the first ones and, as a consequence, an increased incidence of chronic diseases such as tumors has been observed [36,37,38,39,40,41]. It is well-known that macrophages and other cells produce nitric oxide and prostaglandins and that between these two components there is a cross-talk. In fact, both NO and PGs are blocked by nitric oxide synthase-inhibitors (NOS-inhibitors), but this effect can be suppressed through coincubation with L-arginine, which is the precursor of nitric oxide. Furthermore, inhibition of cyclooxygenase-2 (COX-2) in inflammation determines a change in the L-arginine-nitric oxide pathway, while COX-2 inhibition causes a reduction in NOS effect in human platelets [42]. In vivo experiments [42] show an anti-inflammatory effect of cinnamon aqueous extract in a mouse model of paw edema similar to indomethacin, a drug acting as a strong inhibitor of COX-2. As observed, edema reaches the maximum volume in its third phase, when there is infiltration of neutrophils and the release of free radicals from them. Cinnamic aldehyde, contained in cinnamon, impeded neutrophils’ infiltration of the edema, determining, consequently, a decrease in inflammation. Furthermore, it is clear that myeloperoxidase (MPO), an enzyme contained in azurophilic granules of neutrophils, is the molecule that causes tissue damage. There is evidence of an effect of cinnamon extract in inhibiting MPO, preventing tissue injury [43]. As far as the role of chronic inflammation in the pathogenesis of cancer, evidence about the ability of cinnamon extract to inhibit inflammatory molecules could be strong encouragement for scientists to go on in this field of research because of the real effects that have been reached with this substance not only in vitro, but also in in vivo models.

### 2.4. Inhibition of PI3K/Akt/mTOR, MAPK-P38alfa and DHFR

PI3K/Akt/mTOR signals, promoted by point mutations of PI3K and Akt and by inactivity of the phosphatase and tensin homolog (PTEN), play an important role in the regulation of the proliferation and apoptosis of cancer cells, especially from ovarian, gastric and breast neoplasms [44,45,46]. In detail, an irregular function of PI3K/Akt/mTOR determines an increased expression of fusin, which subsequently stimulates C-X-C chemokine receptor type 4 (CXCR-4)-related STAT3; this cascade of signals allows the maintenance of stemness in cancer cells [47]. Experiments with oral cancer cells exposed to aqueous cinnamon extract allowed the detection of an inhibitory action of the natural ingredients of this compound on cellular growth due to the fact that it down-regulates different molecules of the PI3K/Akt/mTOR pathway, preventing its activity [6]. Obviously, more and more evidence is needed before declaring that the programmed death of cells observed is due to an up-regulation of the PI3K/Akt/mTOR pathway from compounds in cinnamon extract, but the experiment cited surely represents a starting point. MAPKP-38α is a kinase activated by pro-inflammatory molecules, oxidative stress and heat shock, all mechanisms involved in tumorigenesis [48,49,50,51,52,53,54,55]. Various evidence [9] exists about the action of cinnamon compounds as a substrate for MAPKP-38α, preventing the bond of the “real” substrate and, consequently, blocking the function of this pathway, including growth and survival stimulation [6]. Other evidence comes from dihydrofolate reductase (DHFR), a protein that induces the transformation of dihydrofolate in tetrahydrofolate, which is finally the cofactor in the synthesis of different molecules fundamental in cell proliferation such as thymidylate, purines and some amino acids [15]; for this reason, DHFR has been used as a target for drugs in the treatment of various tumors, such as with methotrexate [16]. The mechanism of action for aqueous cinnamon extract is the same as that for MAPKP-38α: it ties as a substrate to DHFR, preventing the bond of its “real” substrate and, consequently, blocking its proliferative stimulation of cancer cells. This evidence could indicate a possible effect of cinnamon extract as an anti-cancer molecule due to the fact that it is capable of inhibiting the activity of MAPK-P38alfa and dihydrofolate reductase, molecules which normally have crucial roles in the proliferation and survival of neoplastic cells. In detail, aqueous cinnamon extract interacts with catalytic residues of MAPKP38α and DHFR, such as Val38 and Tyr35, for the first and with Ala9 and Tyr121 for the second, blocking or significantly modifying their function [6].

### 2.5. Inhibition of Angiogenesis

**Figure 2:**
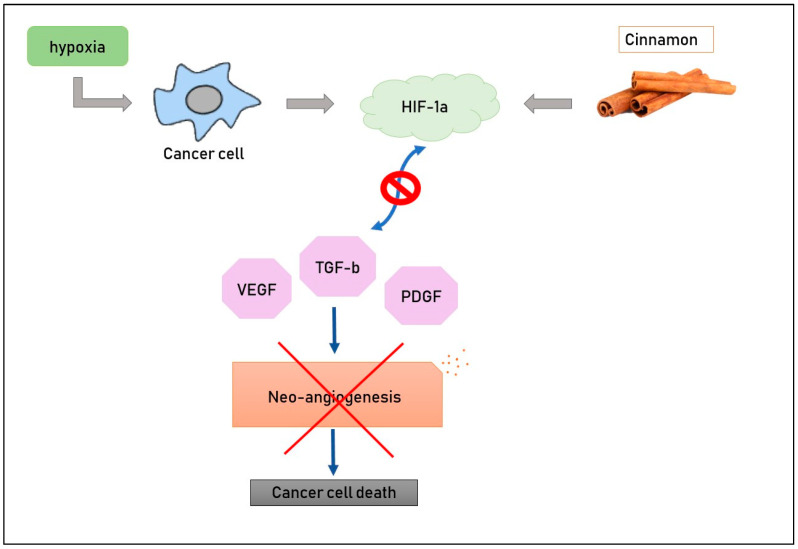
Cinnamon compounds down-regulate levels of HIF-1a, with the results being a decrease in pro-angiogenic factors and, consequently, of new blood vessel formation.

The effect of aqueous cinnamon extract has been studied in a mouse melanoma model [17] in order to evaluate its possible action in inhibiting angiogenesis. It is well-known that cancer cells need more and more nutrients to grow, and these compounds come from blood, so it is crucial for tumors to have new blood vessels in their macroenvironment. Hypoxia and pro-angiogenic molecules such as vascular endothelial growth factor (VEGF), platelet-derived endothelial growth factor (PDGF) and transforming growth factor beta (TGF-b) constitute the main stimuli for neo-vascularization. Scientists treated mouse melanoma cells with cinnamon extract at various doses (0.3 or 0.5 mg/mL) and then evaluated the levels of pro-angiogenic molecules; RT-PCR and ELISA methods were used to detect the expression of VEGF, PDGF and TGF-b, and it was demonstrated that aqueous cinnamon extract inhibited these factors both in mRNA and in proteins (Figure 2). Its activity is clinically evident, too, if we pay attention to the dimensions of the spleen and lymph nodes, which are significantly smaller in mice treated with this extract than those in untreated mice [56]. HIF-1a is a transcription factor whose activity depends on tissue oxygen levels, [8,18]. In fact, hypoxia causes an increase in the presence of pro-angiogenic molecules and stimulates neo-vascularization. The administration of cinnamon extract down-regulates levels of HIF1a, conducting a reduction in the formation of new blood vessels. This evidence allows us to state that cinnamon inhibits neo-angiogenesis [56].

## 3. Cinnamon and Hematological Malignancies

**Figure 3:**
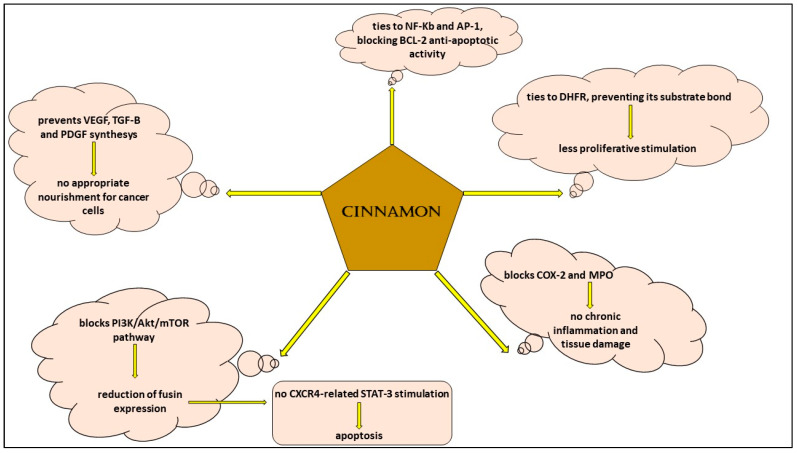
Signaling pathways of aqueous cinnamon extract in hematological malignancies.

The use of medical plants has been demonstrated to be useful in hematological malignancies, and this fact is undoubtedly arousing because every day clinicians in hematology and oncology units are confronted with the adverse effects of traditional drugs, which can sometimes cause the stop of the treatment if it is intolerable for patients and dangerous for their health [57,58,59,60,61]. Scientific research showed the efficacy of different natural compounds both in the prevention and cure of cancer [62,63,64,65,66,67,68,69,70]. Allegra and colleagues [33] studied the effects of rosemary in various types of cancer and put in evidence its action in augmenting antioxidant molecules, decreasing pro-inflammatory cytokines and preventing neo-vascularization, all functions crucial for the insurgence and progression of a tumor in general. In a work, the authors analyzed aqueous cinnamon extract and showed that its mechanisms of action are similar to those of rosemary; in particular, they collected experiences from different scientists and demonstrated that cinnamon extract has a role in hematological malignancies such as leukemia and lymphoma because it is capable of inhibiting the pathways and molecules involved in cellular proliferation: it ties to NF-KB and AP-1, determining a decrease in the correspondent RNA expression and translation and preventing the anti-apoptotic action of Bcl-2 [1]; furthermore, cinnamon extract modifies the function of PI3K/Akt/mTOR, dihydrofolate reductase and COX-2, with the consequences of blocking proliferation cell mechanisms and inflammation cascade signals [6,71,72,73,74,75,76] (Figure 3). However, there are different limits which make us still far from using this compound in everyday practice. For example, a standard method to obtain cinnamon extract in which there are specific doses of its ingredients cinnamic aldehyde and cinnamyl aldehyde does not yet exist; furthermore, the possibility of adverse events due to long-term use and a possible different effect depending on the two tested oral and subcutaneous administration methods has to be clarified.

### 3.1. Acute Myeloid Leukemia

Leukemia is a hematological malignant disease due to abnormal proliferation and differentiation problems of blood cells [77]. Acute myeloid leukemia (AML), in particular, is characterized by damage in precursor cells of the myeloid lineage. It constitutes one-third of all leukemias, even if it is just 1.2% of all new tumors per year in the United States [78,79]. The possible inhibitory effect of aqueous cinnamon extract in cells from hematological malignancies has just been studied in the past, and it seems to be effective [80]. Different studies are putting in evidence regarding the role of the CD45 phosphatase in white blood cells and its mechanism; thanks to this, it could stimulate cellular growth and apoptosis both in normal and leukemic cells. In detail, it is well-known that a correct balance between phosphatase and kinase functions is fundamental for the normal processes of the proliferation of cells [81], in particular for the progression of cell cycles through G0/G1, S and G2/M phases. It was demonstrated that cells of all three lines in G2/M phases augmented in a dose-dependent manner after being exposed to aqueous cinnamon extract [80]. This compound has also been tested in combination with doxorubicin to evaluate both the possible synergic effect in therapy and other aspects such as the reduction of drug toxicity [82]. Doxorubicin is an anthracycline used in the treatment of various kinds of cancers, such as leukemia, and its use is related to adverse effects such as cardio-toxicity and hepatic toxicity [83]. The in vivo study of Bukhari and colleagues [82] starts from the induction of AML in rats; then, animals were divided into two groups: one received the combination treatment of doxorubicin and aqueous cinnamon extract, the other one received just the anthracycline. In order to evaluate the results, pro-proliferative molecules glyceraldehyde-3-phosphate dehydrogenase (GAPDH) and NF-KB components were determined with the real-time PCR method. It was evident that the combination therapy conducted the inhibition of the GADPH and NF-KB components, leading finally to apoptosis of cancer cells and to an improvement of laboratory parameters such as a decrease in white blood cells and a recovery of the normal balance between lymphocytes, monocytes and eosinophils. Furthermore, in rats receiving cinnamon extract, a reduction in hepatic toxicity was observed, suggesting that this compound could be used in combination with doxorubicin to improve anti-leukemia treatment [82].

#### 3.1.1. Acute Promyelocytic Leukemia

Acute promyelocytic leukemia (APL) is characterized by translocation T (15;17), which consists of the translation of retinoic acid receptor gene from chromosome 15 to chromosome 17. This event leads to the production of a fusion gene called PML-RARα which, in turn, causes the blockage of cell maturation at the promyelocytic stage. APL is characterized by severe symptoms of both bleeding and disseminated intravascular coagulation (CID) disease, with an elevated mortality risk. In vitro studies [62] show that cinnamon extract is capable of inhibiting the growth of HL-60 cells, which are promyeloblasts isolated from the peripheral blood of patients with APL and used in scientific research. The easiest method to investigate cell apoptosis consists of the use of Hoechst staining, which puts in evidence differences in nuclear chromatin and, consequently, lets us identify healthy and apoptotic cells through the morphology of the cell nucleus [84,85,86,87,88]. In particular, apoptotic cells appear like small bodies with fragmented and peripheral deoxyribonucleic acid (DNA), while healthy cells are larger and oval with a single and undefined nucleus. DNA fragments of cells in the apoptosis phase are of a blue fluorescent using Hoechst staining; in contrast, the DNA of healthy cells does not saturate with coloring and it appears just like spots at microscopic observation. In detail, it can be stated that after an exposure of 24 h, a dose of 0.01 mg/mL of aqueous cinnamon extract inhibited 74.6% of leukemic cells; after 48 h this was 85.5% and, finally, after 72 h this was 90.1% (Table 1). Furthermore, scientists questioned themselves about the effect of the compound at a different dose and, interestingly, they noticed that increasing the dosage to 2 mg/mL led to a reduction in cytotoxicity at 50.7% after 24 h and at 73.2% after 72 h [62]. This evidence is interesting for hematologists both for the possible effect of aqueous cinnamon extract in contrasting the anti-apoptotic mechanisms of cancer cells and for its proved capacity to induct a strong inhibition of acute promyelocytic leukemia cell lines in vitro.

#### 3.1.2. Acute Erythroblastic Leukemia

Acute erythroblastic leukemia is the M6 type according to FAB classification of acute myeloid leukemias; it is characterized by the presence of K562 cells [89,90,91,92] and, clinically, by fever, severe anemia, hemorrhage, infections an increase in the volume of the liver and spleen. Additionally, thrombocytopenia and leukopenia can be present [89,93]. Kim and his group [43] studied proviral insertion in murine lymphomas-1 (Pim-1), a proto-oncogene with important roles in the proliferation and differentiation of cells, which is overexpressed in erythroblastic leukemia, gastric carcinoma, prostate cancer, bladder cancer and is related to a poor prognosis; it acts via phosphorylating the anti-apoptotic molecules of Bad and preventing apoptosis. Researchers found that aqueous cinnamon extract is capable of inhibiting Pim-1 thanks to the bond of its ingredients to the structure of this kinase; in fact, Pim-1 has a novel hinge region and a unique hinge region, a sequence which links the fragment antigen binding region to the fragment crystallizable region of the protein [43] and to which natural compounds of cinnamon tie, determining apoptosis of cells of erythroblastic leukemia. In detail, seventy-seven kinases have been studied comparing the percentage that cinnamon extract was able to inhibit, and it was demonstrated that Pim-1 was inhibited at least 80%, more than the other molecules tested. The anti-cancer effect can also be observed macroscopically because of the dose-dependent reduction in tumor volume, so we can affirm which aqueous cinnamon extract is a Pim-1 inhibitor and, in the future, a specific drug targeting Pim-1 could be an interesting opportunity in the daily struggle against cancer [43].

### 3.2. Lymphoma

Polyphenols, with which aqueous cinnamon extract is rich, are capable of reducing the in vitro proliferation of lymphoma cells in a dose-related modality [43], but this evidence is not confirmed in vivo. In fact, research about the effect of cinnamon extract in gastric lymphoma Helicobacter pylori (HP)-related infection shows unsatisfying results [94]. Helicobacter pylori (HP) infection is very common worldwide, and it is related to the onset of gastritis, gastric and duodenal ulcers and gastric lymphoma. The cure for the infection includes two or three different drugs such as antibiotics, and it is effective in about 90% of cases. Gastric lymphoma in 90% of cases is due to HP infection; in fact, the eradication of this bacterium often leads to a complete and long-term remission of lymphoma [95,96,97,98,99]. In their in vivo experiment, Nir and colleagues [94] did not have good results in terms of the reduction in colonization rate of HP measured through Urea breath-test (UBT) after a treatment with aqueous cinnamon extract, so we just state that cinnamon extract may be effective at higher doses than that used, or that a reduction in infection rate was reached before measurement, so just after 2 weeks from administration of the compound, considering instead that, in this experiment, the colonization rate was measured after 4 weeks. This evidence gives us the possibility to reflect on the fact that the contrast between in vitro and in vivo results could be overcome by making more measurements of UBT, because it is possible that after two weeks from treatment of patients with gastric lymphoma Helicobacter pylori-related infection with cinnamon extract, a good response in terms of reduction of the infection rate could be observed, and this response could be lost after four weeks; this condition could indicate that adjustments in doses, way or timing of the administration of aqueous cinnamon extract are needed. Obviously, a decrease in UBT and, finally, the eradication of HP are desired because they almost always correspond to a complete and long-term remission of HP-related gastric lymphoma. Furthermore, a surprising effect of aqueous cinnamon extract is the reduction of UBT in patients with very high values at the first screening, so it can be speculated that this compound could be more effective in those who have a severe infection and a consequent gastric lymphoma HP-related infection, giving clinicians another instrument for curing the disease in combination with traditional drugs [94].

## 4. Clinical Trials

There are various clinical trials about the possible use in vivo of cinnamon in different pathologic conditions (Table 2) [9]. The NCT05229029 phase-two clinical trial, started in February 2022, is currently recruiting participants: the aim is to evaluate the improvement in quality of life and fatigue symptoms in patients treated with chemotherapy after oral administration of a traditional Chinese medicine compound (including cinnamon 3 g), proving its capability to reduce a fatigue score characterized by different levels from 0 (no fatigue) to 10 (maximum imaginable fatigue); other factors considered in this trial, with 196 participants as its estimated enrollment, are sleep quality, liver and kidney function and blood routine tests. Moreover, in patients with head and neck cancer treated with radiotherapy or chemo-radiotherapy, the observation trial NCT03738657 used cinnamon together with other substances such as pineapple and banana in odor pens to test the capacity of recognizing a specific smell and, consequently, to establish the damage level caused by radiotherapy to olfactive structures such as the tongue, parotid and salivary glands: in detail, the aim of this study, not completed yet and with an estimated enrollment of 110 participants, is to correlate quantitative chemosensory gustatory function with dosimetric information about the fundamental structures relevant to the perception of taste, density of fungiform papillae, the use of platinum-based therapy, the patient-referred taste loss and the weight to define the nutritional status of the patient. The interventional, randomized study NCT00970541 evaluated the ability of cinnamon aqueous extract to act in the same way as insulin, improving insulin resistance, since it is well-known that the last condition is a crucial risk factor in the onset of gynecological cancer. In this trial, whose recruitment was completed in August 2021, there are two arms: an active comparator in which patients received 500 mg of cinnamon in the form of two capsules before meals three times per day and a placebo comparator in which patients received 500 mg of wheat flour before meals three times per day; the results are not posted yet.

## 5. Conclusions

Hematological malignancies are between the most frequent types of cancer, and they are associated with a high rate of morbidity and mortality [100,101,102,103,104]. Every day, science, on the one hand, gives us new information about processes of pathogenesis and new methods for cures and, on the other hand, screening programs give us the possibility to make a precocious diagnosis, which is crucial for the treatment phase both for patients and for clinicians. Drugs commonly used in hematological malignancies consist of chemotherapy, which often causes adverse effects [105]; for example, doxorubicin is characterized by cardiotoxicity due to autophagy mechanisms, which are normally a defense instrument for cells but are dysregulated and excessive in this case [106,107]. It is well-known that different risk factors of cancer exist, such as obesity, smoking, excessive consuming of alcoholic drinks, little physical activity and an incorrect diet, but unmodifiable factor risks also exist and they depend on individual genetic traits. A fundamental goal for those who cure hematological patients is not only an extension of lifetime but also an improvement of quality of life. Speaking of which, various foods from the vegetal world contain polyphenols, antioxidant molecules associated with anti-inflammatory and anticancer effects. Scientific research demonstrates that these compounds inhibit the onset and progression of tumors, both in vitro and in vivo [108]. Furthermore, oxidative stress causes genetic modifications in nucleic acids, preventing, for example, apoptosis, and this condition is undoubtedly associated with an augmented risk of carcinogenesis. Different natural molecules, such as cinnamon extract in its aqueous form, are demonstrated to be effective in cancer and they act at various levels, such as reducing the oxidative stress of cells or upgrading the activity of the immune system in recognizing tumor cells as non-self [33,51,53,55,62]. Globally, cinnamon extract stimulates apoptosis through an augmentation of the expression of NF-KB and AP-1 [1], but also through determining the activation of cytotoxic CD8+ T cells [9]; moreover, it prevents the proliferation of cancer cells thanks to the inhibition of COX-2 and myeloperoxidase [43], molecules normally involved in inflammation and tissue damage that, as it is well-known, are at the basis of carcinogenesis. Various pathways represent a target for the compounds which constitute aqueous cinnamon extract, such as PI3K/Akt/mTOR and MAPK-P38alfa [6,109], normally implicated in cell proliferation. However, its activity is also related to neo-angiogenesis; in fact, cinnamon extract inhibits the synthesis of VEGF and other pro-vascularization factors [62,110,111,112], blocking the useful network of vessels necessary for cancer cell survival. In conclusion, this work does not authorize us to state that aqueous cinnamon extract is a cure for cancer, but the scientific evidence we collected shows that it could in future represent a valid help for clinicians in combination with traditional treatments in order to strongly attack tumors. Obviously, research has to clear the dose at which aqueous cinnamon extract has to be used, the quantity of compound that is safe to use in order to prevent adverse effects and the mechanisms through which it is capable of modifying the microenvironment and macroenvironment of tumors, blocking the proliferation and survival of cancer cells. The possibility of using cinnamon extract in clinical practice is discussed; even if its use could appear very interesting, more studies are necessary to define the real potentiality of this substance in cancer. The results we presented in this review represent just a starting point for more and more future studies, with the aim of first collecting more pre-clinical information about the possible use of aqueous cinnamon extract in cancer, then translating it into in vivo experiments and finally, if its efficacy is confirmed, letting this natural compound become part of daily clinical practice together with traditional drugs in the treatment of cancer and, in particular, of hematological malignancies. 

## Figures and Tables

**Table 1:** Percentage of leukemic cell inhibition based on time from exposure to a dose of 0.01 mg/mL of cinnamon aqueous extract.

**Exposure time (h)**	24	48	72
**Cell inhibition (%)**	74.60	85.50	90.10

**Table 2:** Clinical trials about the use of cinnamon in different pathologic conditions ^1^.

Study Title	Conditions	NCT Number
Improving Insulin Resistance in Gynecological Cancer Patients	Insulin Resistance	NCT04139694
A Dose Escalation Study of the Safety and Pharmacokinetics of GSK1363089 (Formerly XL880) Administered Orally to Subjects with Solid Tumors	Solid Tumors	NCT00742131
RSYR for Fatigue Reduction in Cancer Fatigue Caused by Chemotherapy	Cancer-related Problems/Conditions	NCT05229029
Gustatory Function Following Radiotherapy to the Head and Neck	Head and Neck Cancer	NCT03738657
The Effect of Cinnamon on Ovulation Induction in Women with Polycystic Ovary Syndrome	Polycystic Ovary Syndrome	NCT03778099
Cinnamon Extract on Menstrual Cycles in PolyCystic Ovary Syndrome (PCOS)	Polycystic Ovary Syndrome	NCT01483118
The Effect of Cinnamon Extract on Insulin Resistance Parameters in Polycystic Ovary Syndrome: A Pilot Study	Polycystic Ovary Syndrome	NCT00331279
Effect of Cinnamon Extract on Insulin Resistance in Polycystic Ovary Syndrome	Polycystic Ovary Syndrome	NCT00970541

^1^ Data available at https://clinicaltrials.gov (accessed on 18 February 2023) [9].

## Data Availability

Not applicable.
